# LATE-LIFE MORTALITY GWAS IN FLIES IDENTIFIES DIABETES AND OBESITY REGULATED TO REGULATE MORTALITY AND RESILIENCE

**DOI:** 10.1093/geroni/igac059.1737

**Published:** 2022-12-20

**Authors:** Tyler Hilsabeck, Sudipta Bar, Kenneth Wilson, Jennifer Beck, Christopher Nelson, Rachel Brem, Pankaj Kapahi

**Affiliations:** Buck Institute, Santa Rosa, California, United States; Buck Institute for Research on Aging, Novato, California, United States; Buck Institute for Research on Aging, Novato, California, United States; Buck Institute, Novato, California, United States; Buck Institute, Novato, California, United States; University of California at Berkeley, Novato, California, United States; Buck Institute for Research on Aging, Novato, California, United States

## Abstract

Variations in rate of aging in geneticaly heterogenious populations supports the hypothesis that aging is at least partially genetically regulated. However, genetically identical individuals also vary in their time of death. We have observed in D. melanogaster that this variation is genotype-dependent, as specific genotypes have characteristic survival curve shapes that are largely reproducible. Typical aging studies reduce a strain’s lifespan down to a population-level value, i.e. mean lifespan. While these metrics can represent the trends in a population, they are unable to encapsulate the variation in the aging of individuals from the same distinct population. Instead, we used two values that characterize the logistic fit of a strain’s mortality late in life: the risk of initial mortality (α) and the rate of aging (β). To identify regulators of the rate of aging, we performed a Genome-Wide Association Study (GWAS) of β for 160 different fly strains from the DGRP collection on two different diets late in life. This approach identified the candidate gene Diabetes and Obesity-Regulated (DOR), which has known roles in stress response, autophagy, and senescence, as having a role in the late-life mortality. DOR inhibition leads to a significant increase in late-life mortality that is preceded by a reduction in healthspan-related traits. Further, germline-specific inhibition is sufficient to increase senescence-related factors and shorten lifespan. We conclude that a decrease in DOR, a conserved gene, compromises an organism’s resilience through increased inflammation, senescence, and increased mortality, providing a potential target for bolstering the decline seen in human aging.

